# Oligomerization Function of the Native Exon 5 Sequence of Ameloblastin Fused with Calmodulin

**DOI:** 10.1021/acsomega.4c07953

**Published:** 2025-02-20

**Authors:** Monika Zouharova, Petr Herman, Lucie Bednarova, Veronika Vetyskova, Romana Hadravova, Klara Postulkova, Lucie Zemanova, Jiri Vondrasek, Kristyna Vydra Bousova

**Affiliations:** † Institute of Organic Chemistry and Biochemistry of the Czech Academy of Sciences, Flemingovo namesti 5/542, 16000 Prague, Czech Republic; ‡ Second Faculty of Medicine, Charles University, V Úvalu 84, 15006 Prague, Czech Republic; § Faculty of Mathematics and Physics, Charles University, Ke Kralovu 5, 12116 Prague, Czech Republic; ∥ Faculty of Science, University of Hradec Kralove, Rokitanskeho 62, 500 03 Hradec Kralove, Czech Republic

## Abstract

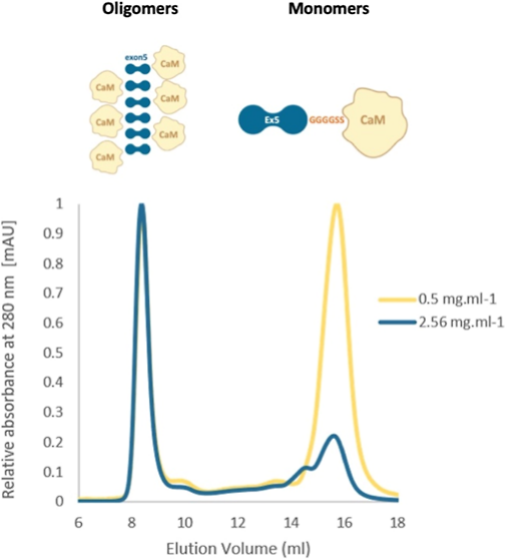

The evolution of proteins is primarily driven by the combinatorial assembly of a limited set of pre-existing modules known as protein domains. This modular architecture not only supports the diversity of natural proteins but also provides a robust strategy for protein engineering, enabling the design of artificial proteins with enhanced or novel functions for various industrial applications. Among these functions, oligomerization plays a crucial role in enhancing protein activity, such as by increasing the binding capacity of antibodies. To investigate the potential of engineering oligomerization, we examined the transferability of the sequence domain encoded by exon 5 (Ex5), which was originally responsible for the oligomerization of ameloblastin (AMBN). We designed a two-domain protein composed of Ex5 in combination with a monomeric, globular, and highly stable protein, specifically calmodulin (CaM). CaM represents the opposite protein character to AMBN, which is highly disordered and has a dynamic character. This engineered protein, termed eCaM, successfully acquired an oligomeric function, inducing self-assembly under specific conditions. Biochemical and biophysical analyses revealed that the oligomerization of eCaM is both concentration- and time-dependent, with the process being reversible upon dilution. Furthermore, mutating a key oligomerization residue within Ex5 abolished the self-assembly of eCaM, confirming the essential role of the Ex5 motif in driving oligomerization. Our findings demonstrate that the oligomerization properties encoded by Ex5 can be effectively transferred to a new protein context, though the positioning of Ex5 within the protein structure is critical. This work highlights the potential of enhancing monomeric proteins with oligomeric functions, paving the way for industrial applications and the development of proteins with tailored properties.

## 1 Introduction

Protein engineering is a rapidly advancing field focused on the design and modification of proteins to create new functions or enhance existing ones.^1^ The field encompasses several approaches: rational design, directed evolution, and computational *de novo* methods.^2^ Protein engineering holds significant promise for the development of new therapeutics, biomaterials, and industrial applications.^1^ Among various strategies in protein engineering, inducing oligomeric functions in natively monomeric proteins presents a particularly intriguing approach. One lesser-explored strategy in protein engineering involves inducing oligomerization in proteins that are naturally monomeric, thereby increasing the local protein concentration to enhance its functionality. Oligomerization refers to the process by which multiple protein subunits assemble into a larger protein complex.^3−6^ Larger proteins or complexes are often preferred over smaller ones due to their increased resistance to denaturation and degradation, resulting from reduced solvent exposure.^7,8^ Additionally, the presence of multiple active sites can improve the functional efficiency. For instance, large multienzyme complexes, like RNA polymerase, exhibit higher turnover rates compared to their individual subunits acting independently.^7^ While these benefits could theoretically be achieved with larger single proteins, nature frequently employs smaller subunits to achieve similar effects. This approach provides a safeguard against translation errors as large protein complexes are formed from smaller monomeric units. The design of artificial self-assembling protein complexes has become a major focus in protein engineering.^9−12^ Diverse techniques have been developed, including the creation of fusion or split proteins and domains, the construction of nanoscaled protein blocks,^10^ and assembly bridging through metal ions,^13^ cofactors,^14^ or disulfide bonds.^15−18^

One of the most established approaches for engineering novel protein oligomerizing molecules is the use of coiled-coil domains, which are helical protein motifs with a strong propensity to form dimers or higher-order oligomers.^19^ Another common approach involves leucine zippers, protein domains that mediate oligomerization through specific interactions among leucine residues.^20^ The primary motivation for developing artificial oligomers is to explore novel structural and functional spaces that are not readily accessible in nature, with the goal of creating innovative solutions for technological, medical, and scientific applications, including protein scaffolds, drug delivery systems, and biosensors. Although engineering protein oligomerization presents considerable challenges, a rational approach is to associate the newly identified oligomerization sequence with a folded, stable, and well-characterized protein. In our project, calmodulin (CaM) serves as a stable framework. Although engineering protein oligomerization poses considerable challenges, a rational approach is to associate the newly identified oligomerization sequence with a simply folded, stable, and well-characterized protein. In our project, calmodulin (CaM) serves as a stable framework.

**Figure 1:**
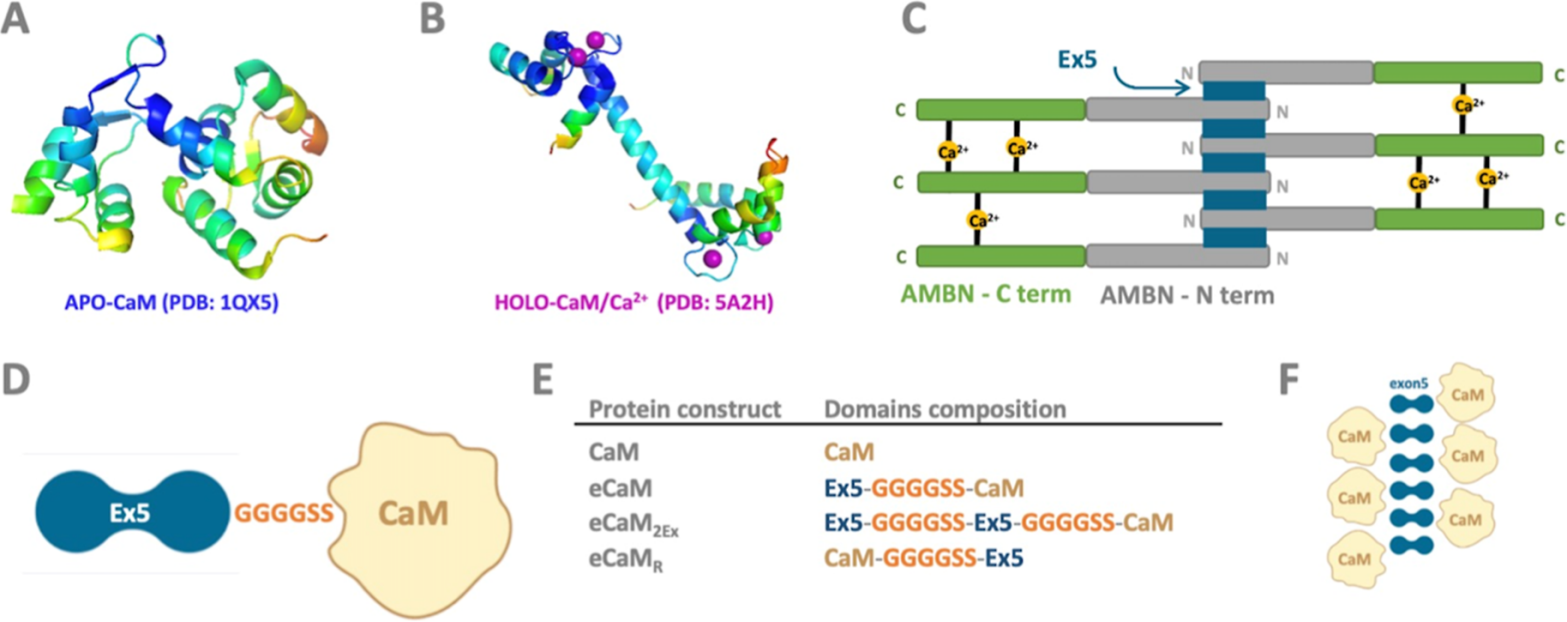
eCaM fusion protein components and design. (A) Apo-CaM structure (PDB: 1QX5), (B) CaM upon Ca^2+^ binding (PDB: 5A2H), (C) schematic representation of AMBN oligomers with N- (gray) and C-termini (green), and Ex5 (blue) responsible for protein oligomerization; calcium ions bridge the C-termini of AMBN monomers (yellow circles),^36^ (D) schematic representation of eCaM composed of N-terminal Ex5 (exon5 encoded sequence, blue), flexible glycine linker GGGGSS, and C-terminal globular CaM (yellow), (E) table displaying all designed fusion protein compositions, and (F) schematic representation of potential eCaM oligomer formation (Ex5 blue, CaM yellow).

As a monomeric globular molecule, calmodulin (CaM) senses changes in cellular Ca^2+^ levels and regulates important cellular processes by interacting with receptors, transporters, and enzymes, and it is involved in gene transcription.^21−24^ CaM typically monitors Ca^2+^ concentration through four canonical EF-hand motifs composed of two α-helices bridged by a loop coordinating bond of Ca^2+^ ions resulting in a 1:4 stoichiometry of CaM/Ca^2+^ interaction.^25,26^ CaM comprises N- and C-terminus globular EF-hand domains connected by a flexible linker.^27−29^ The CaM molecule exhibits significant conformational flexibility, allowing it to adopt a variety of binding modes. These range from compact conformations (where CaM’s central helix bends, and both lobes wrap around the binding site, as shown in Figure 1A^30,31^) to extended conformations (CaM lobes interact with two distinct binding sites, as illustrated in Figure 1B).^32,33^ Interactions between CaM and its target proteins can further alter CaM’s conformation at different Ca^2+^ concentrations, potentially enhancing its Ca^2+^ affinity.^34^ The differences in Ca^2+^ binding affinities and conformational flexibility make CaM a highly dynamic and versatile protein sensor, capable of regulating target proteins across a wide range of Ca^2+^ levels. Most CaM/target complexes described to date are Ca^2+^-dependent; however, several proteins can also interact with Ca^2+^-free CaM through the so-called IQ motif, which has the consensus sequence IQXXXRGXXXR.^35^

In contrast to CaM, meloblastin (AMBN) is an intrinsically disordered protein (IDP) that plays a crucial role in the formation and mineralization of dental enamel. In solution, AMBN assembles into multimeric subunits that form higher-order oligomeric structures (Figure 1C),^36,37^ which are believed to regulate enamel crystal growth and morphology.^38^ Recently, AMBN has been associated with bone development and with the prevention and healing of bone fractures.^39^ Although the specific oligomeric state of AMBN is not fully understood, it is thought to form higher-order structures facilitated by a short 37-amino acid sequence at the AMBN N-terminus, encoded by exon5 on the transcript level (Ex5), which interacts with amelogenin^40^ and also binds to lipid membranes.^41−43^ Studies have demonstrated that the oligomeric form of AMBN can influence the size, shape, and orientation of enamel crystals, as well as the overall structure of the enamel matrix.^44−47^ Specifically, AMBN oligomers can act as templates for enamel crystal formation, promoting crystal growth in specific directions and preventing crystal aggregation.^44^ Deletion of Ex5 results in a complete loss of AMBN’s ability to form oligomers, leading to structural abnormalities in the formation of dental enamel and resulting, for example, in amelogenesis imperfecta.^41^

In this project, we designed and produced a synthetic fusion protein, eCaM, derived from Ex5 of AMBN and the well-characterized monomeric protein CaM. The eCaM construct was developed to investigate the potential for self-assembly of the originally monomeric CaM, driven by the oligomerization sequence of Ex5. We investigated the induced oligomeric states of eCaM, which resulted in various oligomer populations. The fusion protein was analyzed to determine its molecular size and shape, as well as its interactions with a peptide ligand derived from the native binding partner of CaM, the transient receptor potential melastatin-4 (TRPM4) channel. We present the biochemical and biophysical characterization of this novel oligomerizing eCaM fusion molecule, demonstrating the transferability of Ex5’s original capacity to induce protein oligomerization.

## 2 Materials and Methods

### 2.1 Design and cDNA Production of the eCaM DNA Construct

cDNA sequences encoding CaM (UniProt: P0DP26), eCaM, eCaM F67G, eCaM2Ex, and eCaMR were synthesized by GenScript (Piscataway, NJ, USA).

#### 2.1.1 eCaM Construct

In the eCaM construct, CaM is located at the C-terminus of the fusion protein, connected via a flexible linker (GGGGSS) to the N-terminally positioned AMBN-exon5 short sequence, termed Ex5 (UniProt: Q9NP70, AMBN_HUMAN; sequence: 62YSRYGFGKSFNSLWMHGLLPPHSSLPWMRPREHETQQ98).

#### 2.1.2 eCaM2Ex Construct

In the eCaM2Ex construct, a double Ex5 sequence (UniProt: Q9NP70, AMBN_HUMAN; sequence: 62YSRYGFGKSFNSLWMHGLLPPHSSLPWMRPREHETQQ98) is positioned at the N-terminus of the fusion protein. The two Ex5 sequences are linked by a flexible linker (GGGGSS), which is also used to connect the N-terminal double Ex5 to CaM at the C-terminus.

#### 2.1.3 eCaMR Construct

In the eCaMR construct, CaM is located at the N-terminus of the fusion protein and connected via a flexible linker (GGGGSS) to the C-terminally positioned Ex5 sequence (UniProt: Q9NP70, AMBN_HUMAN; sequence: 62YSRYGFGKSFNSLWMHGLLPPHSSLPWMRPREHETQQ98).

The cDNA of these constructs was cloned by standard methods into the pET28_b vector (Piscataway, NJ, USA) for in vitro studies.

### 2.2 Peptide Synthesis

The Ex5 peptide, derived from AMBN (UniProt: Q9NP70·AMBN_HUMAN, positions Y62-Q98), was synthesized using solid-phase peptide synthesis with the standardized Nα-Fmoc protocol.^48^ The purity and identity of the Ex5 were determined using an Agilent 1260 HPLC (Agilent Technologies, Santa Clara, CA, USA) coupled to an ESI-TOF Agilent 6530 mass spectrometer (Agilent Technologies, Santa Clara, CA, USA) with Agilent Jet Stream technology.

### 2.3 Protein Expression and Purification

CaM, eCaM, and its variants (eCaM F67G, eCaM_R_, eCaM_2Ex_) were expressed in *E. coli* BL21 by 0.5 mM IPTG induction at 25 °C. The cells were incubated for 18 h. After harvesting, the cell suspension was resuspended in 50 mM Tris-HCl (pH 7.5), 500 mM NaCl, and 20 mM imidazole, and then disrupted by sonication. The soluble fraction was supplemented with 8 M urea and loaded on a Chelating Sepharose Fast Flow (GE Healthcare, Chicago, IL, USA) charged with Ni^2+^ ions. The proteins were eluted with 50 mM Tris-HCl (pH 7.5), 600 mM NaCl, 8 M urea, 600 mM imidazole, and then renatured by dialysis in 50 mM Tris-HCl (pH 7.5) and 500 mM NaCl. Ca^2+^ contamination was chelated by the addition of 10 mM EGTA for 20 min. Finally, the proteins were subjected to analytical size-exclusion chromatography (ASEC) on Superdex 200 Increase 10/300 GL (GE Healthcare, Chicago, IL, USA) equilibrated with 10 mM tris (pH 7.5), 100 mM NaCl.

### 2.4 Analytical Size Exclusion Chromatography

Analytical size exclusion chromatography (ASEC) of the proteins was performed using an Acta Pure FPLC system (GE Healthcare, Uppsala, Sweden) with a Superdex 200 Increase 10/300 GL column (GE Healthcare, Uppsala, Sweden). The column was equilibrated with a buffer containing the following: 10 mM Tris-HCl (pH 7.5) and 100 mM NaCl and calibrated with a Gel Filtration Standard (Bio-Rad, CA, USA). The concentration of the analyzed proteins ranged from 0.5 to 5.7 mg/mL. The experiments were conducted at a flow rate of 1 mL/min.

### 2.5 Analytical Ultracentrifugation

Sedimentation velocity measurements were performed using an Optima AUC analytical ultracentrifuge (Beckman Coulter, Brea, CA, USA). The eCaM samples were measured in a buffer containing 10 mM Tris-HCl (pH 7.5) and 100 mM NaCl at two concentrations: 0.77 and 2.40 mg/mL. The sedimentation velocity experiments were conducted at 20 °C and 15,000 rpm using double sector cells and an An50-Ti rotor. A total of 1000 scans were recorded at 280 nm for the eCaM sample at 0.77 mg/mL and at 295 nm for the eCaM sample at 2.4 mg/mL, with 1 min intervals between scans. Buffer density, buffer viscosity, and protein partial specific volumes were estimated using Sednterp v3.^49^ The data were analyzed with Sedfit v16.36,^50^ employing the continuous sedimentation coefficient distribution c(s) model for the eCaM sample at 2.4 mg/mL and the continuous sedimentation coefficient distribution c(s) with a bimodal *f*/*f*0 model for the eCaM sample at 0.77 mg/mL, resulting in separate *f*/*f*0 values for monomeric and oligomeric states. The figure was prepared using GUSSI.^51^

### 2.6 Circular Dichroism Spectroscopy

The circular dichroism (CD) measurements were performed using a Jasco-1500 spectropolarimeter equipped with a Peltier thermostated holder PTC-517 (JASCO, Easton, MD, USA). Spectra were recorded at 25 °C in the far-UV range (195 nm −280 nm) with the following experimental setup: 0.5 mm rectangular quartz cell, standard instrument sensitivity, 1 nm bandwidth, a scanning speed of 10 nm/min, a response time of 8 s, and one accumulation. After baseline subtraction, the final data were expressed as molar ellipticities θ (deg·cm^2^·dmol^–1^) per residue. The eCaM samples were prepared in a buffer containing 10 mM Tris-HCl (pH 7.5) and 100 mM NaCl, with protein concentrations of about 50 μM. The numerical analysis of secondary structures was performed using the CD Pro software package, specifically the CONTIN program.^52^

### 2.72.7 Fluorescence Anisotropy Measurements

Steady-state fluorescence anisotropy (FA) measurements were performed using a photon counting spectrometer K2 (ISS Inc., Champaign, IL, USA) following our previously established protocol.^53^ The samples were investigated for CaM affinity to TRPM4np including eCaM, Ex5, and CaM alone in the buffer composed of 10 mM Tris (pH 7.5) and 100 mM NaCl with addition of either 10 mM CaCl_2_ or 10 mM EDTA in order to investigate calcium dependency of forming interactions. The protein samples were kept at about a 50 μM concentration.

### 2.8 Transmission Electron Microscopy

eCaM and CaM at a concentration of 2.6 mg/mL were analyzed in 10 mM Tris-HCl (pH 7.5) and 100 mM NaCl buffer using negative stain transmission electron microscopy (TEM). Parlodion-carbon-coated grids were floated on top of a 5 μL drop of the sample for 5 min. Subsequently, the grids were transferred onto a drop of 2% uranyl acetate, stained for 2 × 15 s, and then dried. Photomicrographs were taken with a JEOL JEM-1011 electron microscope (Peabody, MA, USA) operated at 80 kV.

## 3 Results

### 3.1 Design of eCaM Fusion Proteins

The eCaM fusion protein constructs were designed to explore potential oligomerization functions and macromolecular properties in comparison to the natively monomeric, globular CaM.^54^ The design of eCaM included the Ex5 sequence at the N-terminus, a GGGGSS flexible linker connecting the Ex5 and CaM domains, and CaM positioned at the C-terminus (Figure 1D). The placement of Ex5 at the N-terminus of the fusion protein was chosen based on its native position in AMBN ISO I. The flexible linker was incorporated to provide maximal conformational flexibility and independence for Ex5 and CaM, given their differing characteristics. The primary goal of the study was to determine whether the oligomerization function of Ex5 could be transferred to a chimeric molecule consisting of the natively monomeric and predominantly globular CaM. To enhance the oligomeric properties of the chimera, we developed the eCaM_2Ex_ fusion protein containing two Ex5 sequences at the N-terminus (Figure 1E). Additionally, to evaluate the impact of domain order on oligomerization, we designed eCaM_R_, in which CaM is positioned at the N-terminus and Ex5 is positioned at the C-terminus of the fusion protein.

### 3.2 CaM in eCaM Fusion Protein Gains a New Function

**Figure 2:**
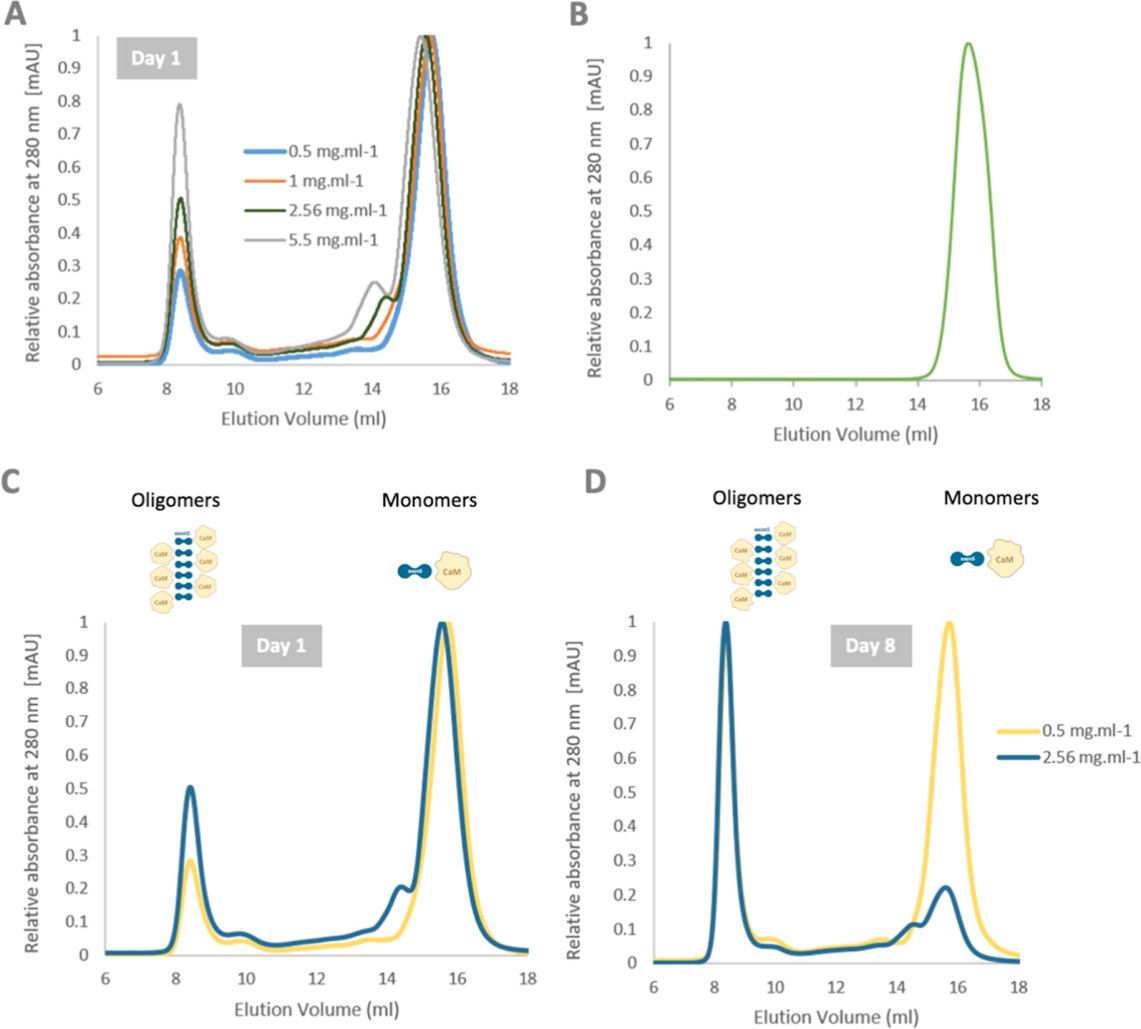
ASEC chromatogram profiles of eCaM monomers and oligomers. (A) eCaM at concentration ranges 0.5–5.5 mg/mL on day 1 after protein purification, (B) CaM alone, and (C) eCaM at 0.5 and 2.56 mg/mL concentrations on day 1 and (D) day 8. All protein batches were eluted on a Superdex 200 Increase 10/300 GL column on the Acta Pure facility.

The oligomerization potential of eCaM was initially assessed using the ASEC method. CaM alone (Mw = 16.8 kDa) served as a benchmark for a monomeric protein. Based on its amino acid sequence, the molecular weight of eCaM was expected to be 21.7 kDa. Purely monomeric eCaM was obtained by ASEC in the final purification step after 1 day (D1) of protein purification from *E. coli* cytosol. ASEC analysis indicated that eCaM exhibited oligomerization immediately following purification on D1, and this oligomerization was shown to be concentration-dependent within the range of eCaM concentrations from 0.5 to 5.5 mg/mL (Figure 2A). ASEC analysis of CaM alone confirmed its monomeric state (Figure 2B). Negative control experiments of CaM and Ex5 interactions as separate proteins/peptides were conducted in standard buffer (10 mM Tris–HCl, pH 7.5, and 100 mM NaCl) (Figure S1). The ASEC analysis of a mixture of isolated Ex5 and CaM domains showed two independent protein peaks, ruling out the formation of oligomers by the individual protein units.

### 3.3 Oligomerization Characteristics of eCaM

**Figure 3:**
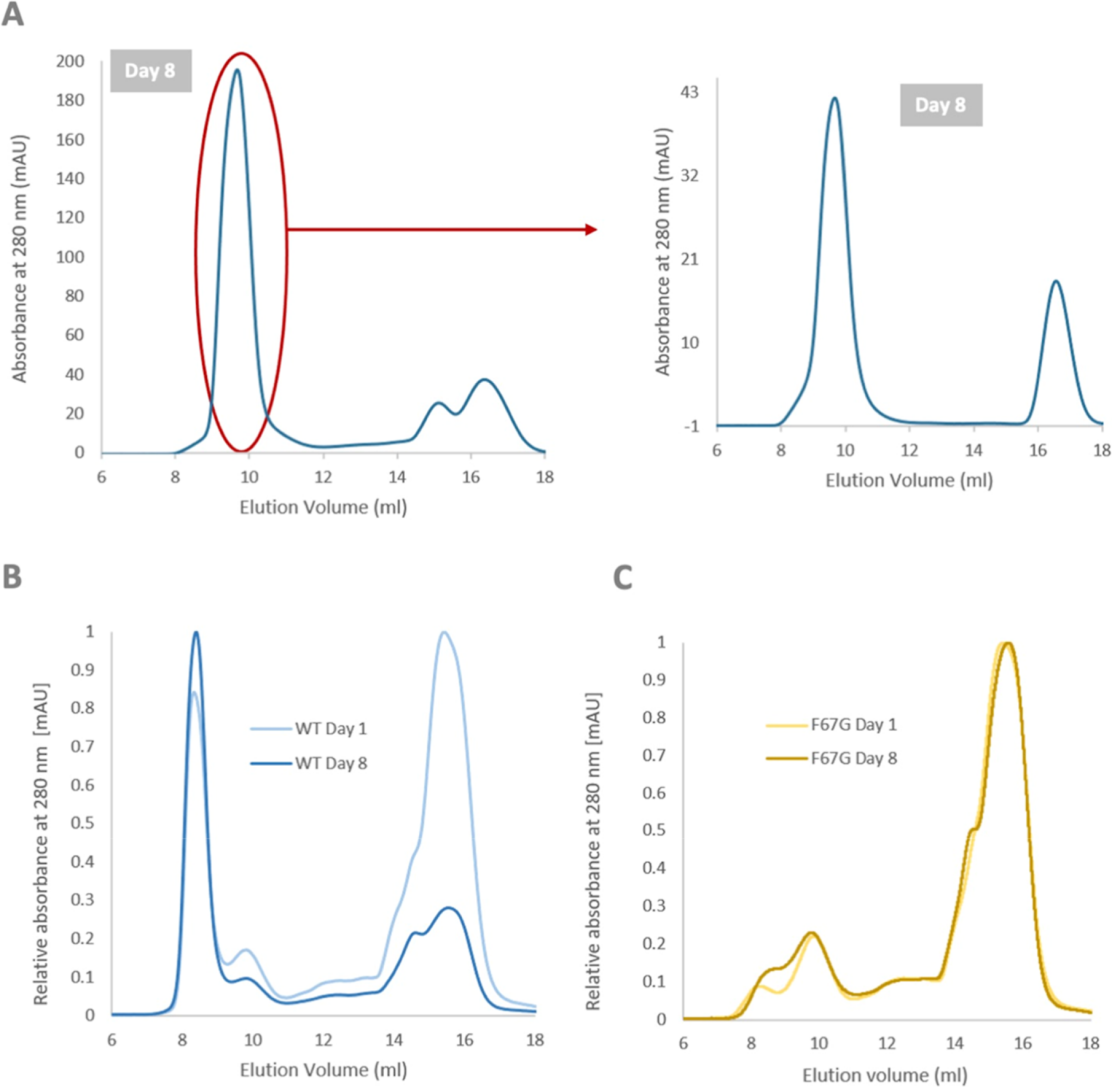
ASEC chromatogram profiles of eCaM oligomers’ disintegration. (A) eCaM at 2.5 mg/mL concentration on day 8 after oligomers (indicated by red oval) separated by ASEC. The oligomers partially disintegrated into monomers proving concentration dependency of the oligomers and monomers equilibrium; (B) eCaM WT at 2.5 mg/mL on days 1 and 8, (C) eCaM F67G mutant at 2.5 mg/mL on days 1 and 8 of protein purification. All protein batches were eluted on a Superdex 200 Increase 10/300 GL column on the Acta Pure facility.

eCaM oligomerization is both concentration- and time-dependent and exhibits reversible behavior. The concentration dependency of eCaM oligomerization was studied over various time scales, revealing that oligomerization increased over time. At higher concentrations (2.56 mg/mL), the protein was nearly completely oligomerized within 8 days at 4 °C. At lower concentrations (0.5 mg/mL), eCaM reached an equilibrium state of monomers and oligomers under the same conditions (Figure 2C,D). Further analysis of the oligomers after 8 days was conducted using ASEC. The eCaM oligomeric fraction from the initial ASEC analysis was subjected to a second round of ASEC analysis after 8 days. Upon dilution, the oligomers separated into a final ratio of approximately 1:2 monomers to oligomers (Figure 3A). ASEC analysis thus confirmed the concentration and time dependency of eCaM oligomerization. To investigate the structural and functional changes induced in eCaM by fusing CaM with Ex5, we conducted detailed biophysical characterizations of eCaM and CaM individually. We examined their molecular size, shape, secondary structure content, and function. Given that CaM is a key calcium-signaling protein, we also assessed how the calcium-binding capacity of CaM is affected by the attachment of Ex5 in eCaM^55^ (see Chapter 3.6).

### 3.4 Ex5 Confirmed as a Sequence That Induces Oligomerization in eCaM

To confirm that Ex5 is an inducer of oligomerization, we prepared the single mutant eCaM F67G, which has previously been shown to disrupt the oligomeric function of the Ex5 sequence in AMBN.^41,42^ ASEC analysis of eCaM wild-type (WT) and eCaM F67G (both at concentration 2.6 mg/mL) showed a complete loss of oligomerization function for the mutant variant (Figure 3B,C). This result supports the role of Ex5 in inducing oligomerization of the eCaM fusion protein. To verify that CaM and Ex5 do not interact with each other as separate domains, we conducted an analysis by ASEC (Figure S1), confirming that the domains behave separately when not linked by a flexible linker sequence.

### 3.5 eCaM Monomers and Oligomers

**Figure 4:**
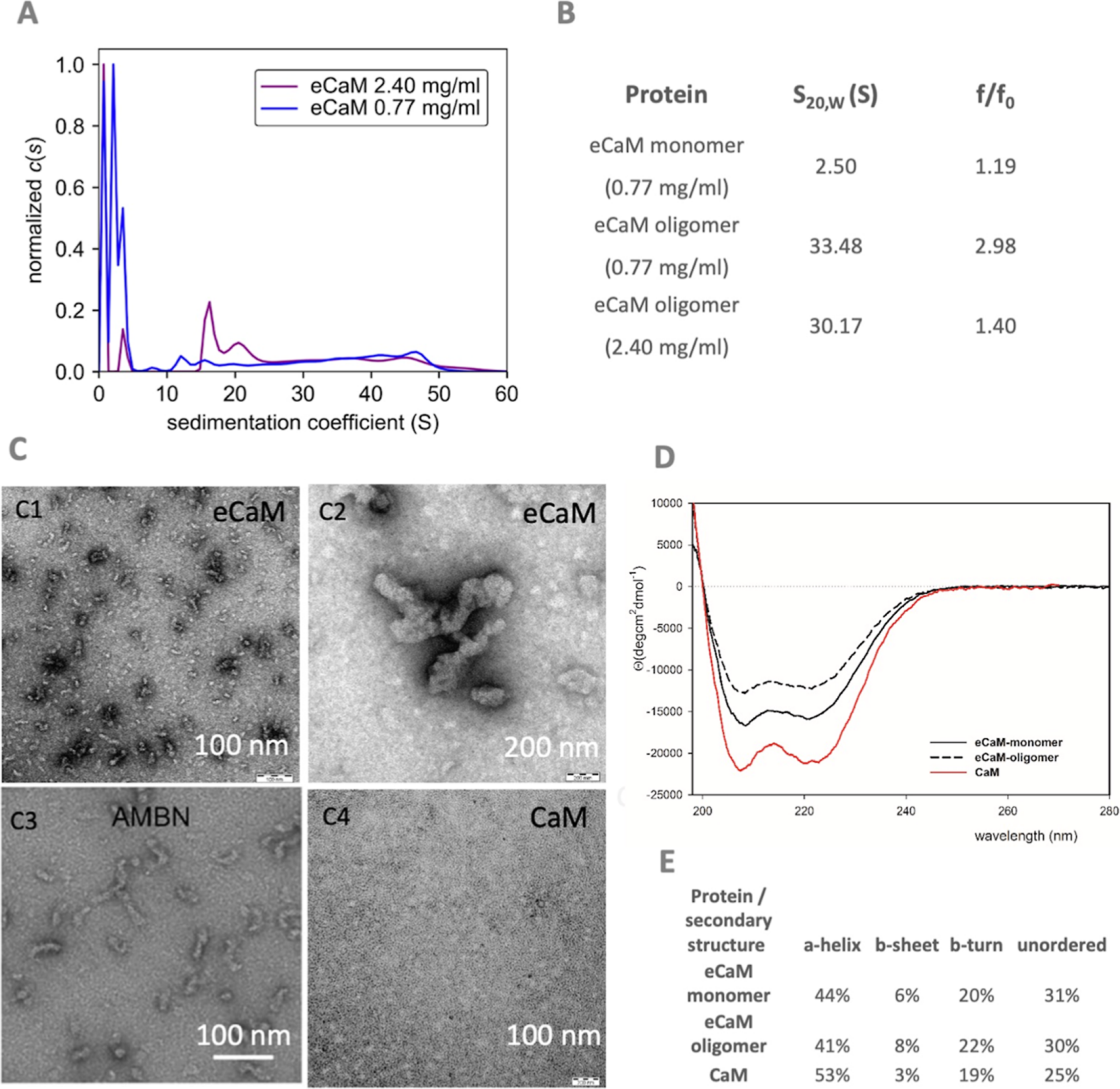
eCaM oligomer analysis. (A) Sedimentation velocity analysis of eCaM at concentrations of 0.77 (blue) and 2.40 mg/mL (violet), shown as continuous size distribution c(s) of the sedimenting species. (B) Summary of sedimentation coefficients (*s*_20,W_) and frictional ratios (*f*/*f*0) derived from sedimentation velocity profiles of eCaM at concentrations of 0.77 and 2.40 mg/mL. (C) TEM of eCaM (C1, C2), AMBN (C3) Wald et al. (2017), and CaM alone (C4) showed similar eCaM oligomeric profile as for AMBN; (D) CD spectra and (E) summarization of secondary structural content of eCaM monomers (black line) and oligomers (black dashed line) and CaM alone (red line).

ASEC analysis demonstrated a concentration-dependent equilibrium between the eCaM monomers and oligomers. To confirm these ASEC data, we employed analytical ultracentrifugation (AUC) techniques. The AUC analysis verified that the eCaM construct exists in an oligomeric state at both lower (0.77 mg/mL) and higher (2.4 mg/mL) concentrations (Figure 4A). However, the oligomerization equilibrium varies between these concentrations, as indicated by the ASEC (Figure 2A). The molecular weight of monomeric eCaM at 0.77 mg/mL, predicted from AUC measurements, was 21.9 kDa, which corresponds well to the expected 21.7 kDa calculated from the protein sequence. The fitted frictional coefficient (*f*/*f*0) of 1.19 indicates a well-folded protein, suggesting a folded structure for monomeric eCaM (Figure 4B). The oligomeric form of eCaM in the 0.77 mg/mL sample exhibited sedimentation coefficients ranging from 6.6 to 52.0 S, with an average of 32.8 S. These values approximately correspond to molecular weights from 526 kDa to 7.28 MDa. The *f*/*f*0 value of the oligomeric fraction was fitted to 2.98, suggesting a more relaxed structure compared with the monomeric form. In the more concentrated eCaM sample (2.4 mg/mL), sedimentation coefficients ranged from 14.5 to 57.7 S, with an average of 30 S. These values approximately correspond to molecular weights from 499 kDa to 2.35 MDa. A smaller peak with an approximate molecular weight of 52.4 kDa was also observed in this sample. The overall frictional ratio (*f*/*f*_0_) of this sample was estimated to be 1.4. This result suggests that eCaM at a concentration of 0.77 mg/mL is more compact compared to the sample at 2.4 mg/mL.

Transmission electron microscopy (TEM) micrographs (Figure 4C) confirmed that eCaM forms oligomers with heterogeneous character, with size limitations in the number of monomers forming the oligomers. This observation is supported by results from the AUC study. Compared to the native oligomerizing AMBN (Figure 4C, part C3), eCaM shows similar oligomer formations, though they exhibit a more compact character due to CaM being a globular protein unit.

The circular dichroism (CD) spectra of eCaM monomeric and oligomeric fractions displayed typical characteristics of α-helical proteins, with two negative peaks at 208 and 222 nm of comparable intensity (Figure 4D).^55^ This indicates that the α-helical structure characteristic of CaM is preserved, albeit with a reduced secondary structural portion. The eCaM oligomeric fraction showed a lower intensity in the CD spectra, suggesting a higher content of β-sheet and β-turn structures typical of oligomeric formations, as confirmed by numerical analysis (Figure 4E). Based on the CD spectra, we hypothesize that CaM, as part of the eCaM fusion protein, retains most of its original secondary structure and remains stable within eCaM oligomers.

### 3.6 Effect of Ex5 on CaM Function in eCaM

**Figure 5:**
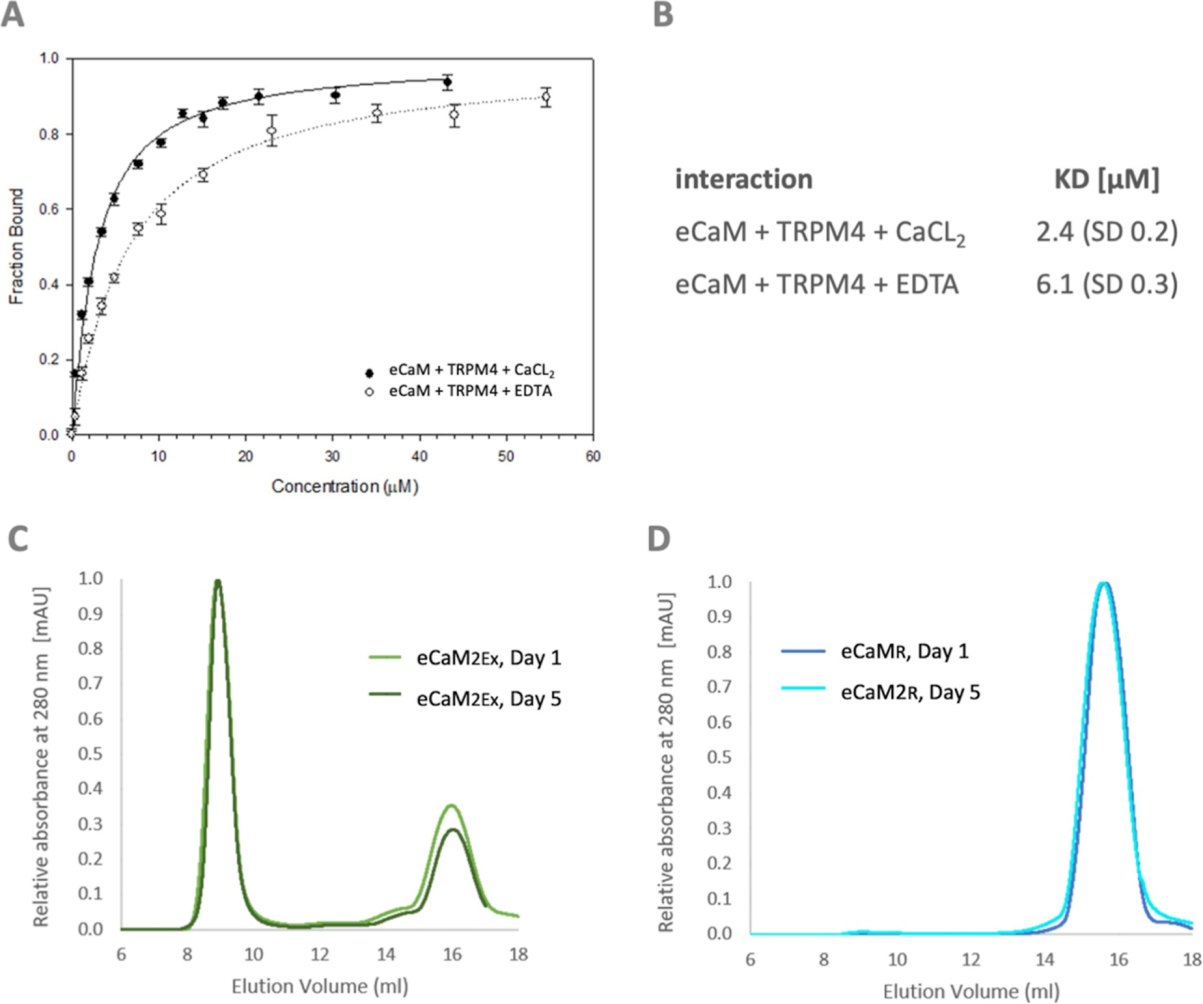
eCaM function analysis. (A) The fraction of TRPM4np bound as a function of eCaM in 10 mM CaCl2 (black line) presence and in 10 mM EDTA (black dashed line) concentrations obtained from steady-state fluorescence anisotropy titrations. Error bars represent the standard deviation of the mean with five independent measurements. (B) Summary of the equilibrium dissociation constants (*K*_D_) of eCaM and TRPM4np complex in the presence and absence of calcium. ASEC chromatogram profiles of eCaM-engineered variants of (C) eCaM_2EX_ and (D) eCaM_R_ at a 2.5 mg/mL concentration on days 1 and 5 obtained by ASEC performed on a Superdex 200 Increase 10/300 GL column at the Acta Pure facility.

To investigate how Ex5 affects CaM function within eCaM, we conducted a binding assay to examine the interaction between CaM and the TRPM4np peptide. The TRPM4np peptide originally comes from the native CaM binding domain present at the N-terminus of the TRPM4 channel.^48^ The binding affinities of TRPM4np in the complex with eCaM were assessed by using a steady-state fluorescence anisotropy (FA) assay. The monomeric form of eCaM was used for this study because it showed a stable behavior of the protein. Carboxyfluorescein-labeled TRPM4np was titrated with eCaM, CaM (as a positive control), and Ex5 (as a negative control). The fluorescence anisotropy value of the peptide was recorded at each titration point. To evaluate the Ca^2+^ dependency of the eCaM/TRPM4np interaction, measurements were performed in buffers supplemented with either 10 mM CaCl_2_ or 10 mM EDTA (without CaCl_2_). The fractions of bound TRPM4np were evaluated for each titration step and plotted against the TRPM4np concentration to determine the equilibrium dissociation constants (*K*_D_) of the studied interactions (Figure 5A,B).

The *K*_D_ value for the eCaM/TRPM4np/Ca^2+^ complex (with 10 mM CaCl_2_) was determined to be 2.4 μM (SD 0.2 μM) and for the apo-eCaM/TRPM4np complex (with 10 mM EDTA) to be 6.1 μM (SD 0.3 μM). Both *K*_D_ values fall within the micromolar range, indicating that eCaM retains its ability to bind TRPM4np. However, the presence of Ca^2+^ enhanced the interaction of the eCaM/TRPM4np complex by approximately 3-fold compared to the Ca^2+^-free environment with EDTA. Additional FA measurement was conducted to evaluate the potential interactions of TRPM4np with the Ex5 peptide as a negative control (Figure S2). The *K*_D_ values for these interactions were not determined (unmeasurably large *K*_D_), confirming that there is no significant interaction potential for the individual protein/peptide components.

### 3.7 Enhancement of the eCaM Oligomerization Rate by a Double Ex5 Sequence

To study the potential increase in the oligomerization rate of eCaM, we designed a new protein fusion named double-Ex5-CaM (eCaM_2Ex_). To determine if the double sequence of Ex5 could enhance eCaM oligomerization, we performed ASEC analysis of eCaM2Ex on day 1 (D1) and day 5 (D5) at a standardized concentration of 2.6 mg/mL (Figure 5C). Most of the eCaM_2Ex_ appeared oligomerized at D1, and this oligomerization remained stable over the next 5 days (D5). In comparison, eCaM analyzed by ASEC at a concentration of 2.6 mg/mL on D1 did not predominantly form oligomers. Although eCaM showed signs of oligomerization, the proportion of oligomers was lower than that of monomers, with the majority of oligomers appearing until 8 days at 4 °C. These observations indicate that the double Ex5 sequence in eCaM_2Ex_ enhances the overall oligomerization process, leading to a higher and more stable oligomerization rate compared to the single Ex5 sequence in eCaM.

### 3.8 Order of Ex5 and CaM in eCaM Does Matter

To determine whether the order of Ex5 and CaM in eCaM influences the oligomerization process, we designed a reversed version of the fusion protein, termed eCaM_R_, in which the Ex5 sequence is positioned at the C-terminus of CaM, using the same linker as that in the original eCaM. ASEC analysis of eCaMR was conducted on days 1 (D1) and 5 (D5) at a standardized concentration of 2.6 mg/mL (Figure 5D). The results revealed that the order of CaM and Ex5 within eCaM is crucial for oligomerization. The analysis showed no oligomerization effect for eCaMR at either D1 or D5, indicating that the placement of Ex5 at the C-terminus of CaM disrupts the oligomerization process. Therefore, we concluded that the order of Ex5 and CaM significantly affects oligomerization, and oligomerization is only viable when Ex5 is positioned at the N-terminus of CaM.

## 4 Discussion

Oligomerization, the process by which proteins assemble into multisubunit complexes, plays a pivotal role in numerous biological pathways, including signal transduction, enzyme regulation, and cellular compartmentalization.^56^ While natural proteins often possess inherent oligomerization functions, the ability to engineer these properties into proteins of interest opens up new avenues for creating tailored functionalities. The presence of a singular protein that can exhibit functional characteristics due to its reversible oligomerization function can be attributed to its exceptional energy utilization efficiency. Furthermore, the temporal progression of the oligomerization process, involving functionally distinct oligomers, may define or guide specific metabolic pathways.^57^ However, under specific conditions, the disintegration of protein oligomers can occur, leading to the release of individual subunits or monomers. Dilution is one such condition that can disrupt protein oligomers. Our study focuses on designing a fusion protein with a newly induced oligomerization function into a naturally monomeric and dynamically stable CaM. By combining the oligomerization motif of AMBN Ex5 and the globular very stable CaM, we aimed to confer the ability for reversible oligomerization based on protein concentration.

Specifically, we have engineered a new two-domain fusion protein to verify whether the Ex5 oligomerization function^41^ is transferable to a completely different protein construct outside its original protein context. Ex5, natively present in AMBN, drives its oligomerization. Thus, we aimed to assess their oligomerization capability within a different protein context. Interestingly, the AMBN is characterized as a fully disordered protein with a highly dynamic character, while CaM is a compact globular rigid protein with very low dynamicity. The selection and comparison of these entirely different contexts—Ex5 as fully disordered and CaM as fully folded—provide a unique opportunity to study whether Ex5 can retain its oligomerization potential in such diverse environments and protein contexts. Furthermore, CaM was chosen for engineering to alter its functional properties, specifically its capacity to bind protein-ligand partners and to influence its structure and folding. This investigation was particularly relevant as it allowed us to explore how Ex5′s oligomerization motif behaves in the context of a protein with different characters, potentially opening new avenues for designing proteins with customizable oligomerization and functional properties.

Native CaM exhibits important cellular functions, as is known to modulate the activity of TRP channels. It participates in the opening^58,59^ and/or closing^60−64^ of these channels and influences downstream or upstream physiological signaling pathways via interactions with TRP binding domains. Numerous peptides mimicking CaM-binding epitopes at different TRP subfamilies have been identified.^48,65−67^ To determine whether the native CaM function to bind the TRP binding domain was preserved in the eCaM construct, we measured the formation of the eCaM/TRPM4np complex previously identified.^53^ The affinity of CaM in eCaM confirmed that the function to bind TRPM4np peptide was preserved (Figure 5A,B). The *K*_D_ of the eCaM/TRPM4np complex is in a similar micromolar range as that of the CaM/TRPM4np.^53^ Surprisingly, the interaction is maintained, even in the absence of calcium. This calcium-free interaction was not reported for the CaM alone/TRPM4np complex.^48^ This result may be explained by changes in the conformation of the CaM molecule, suggesting that its function in eCaM is induced by intermolecular interactions between Ex5 and CaM.

Protein oligomers, composed of two or more subunits, often exhibit modified or completely distinct functional properties compared to their monomeric counterparts.^68^ Their formation results from a delicate balance of intermolecular interactions and structural constraints. Protein concentration can significantly alter the thermodynamic equilibrium between oligomeric and monomeric ensembles.^5,6^ According to the law of mass action, reducing the concentration of the studied fusion protein eCaM shifts the equilibrium toward the dissociated state. It has been proven that eCaM oligomerization is concentration-dependent, resulting in higher oligomer formation at higher eCaM concentrations and disintegration of eCaM oligomers upon dilution. When the oligomerization process is reversible, protein subunits dissociate from the oligomeric assembly as independent entities.^59^ The degree of disintegration is influenced by the dilution ratio, time, and the nature of intermolecular forces stabilizing the oligomers.^69^ This phenomenon warrants detailed study using precise biophysical methods to elucidate the mechanisms underlying the reverse oligomerization process of eCaM. The dynamic equilibrium between monomers and oligomers is crucial in various biological processes, such as the regulation of cellular structures and functions, and in synthetic materials, where reversible binding can lead to responsive or self-healing materials.

The effect of oligomerization on the structure of CaM within eCaM oligomeric formation could not be fully elucidated in this study. Nevertheless, the function of the CaM unit in the eCaM construct is evidently influenced as it retains its ability to bind TRPM4np even under calcium-free conditions. This finding suggests multiple potential mechanisms by which the binding function of CaM could be triggered by the Ex5 peptide. One plausible explanation is that structural changes in the CaM binding site for TRPM4np are induced allosterically. The oligomerization of eCaM likely leads to changes in the CaM structure and consequently its function, highlighting the complex interplay among protein structure, oligomerization, and function.

In this study, we engineered an oligomeric fusion protein composed of Ex5 from AMBN and globular protein CaM. Analytical techniques, including ASEC and AUC, demonstrated the formation of distinct oligomeric and monomeric states in response to varying conditions. Additionally, biophysical studies using CD and fluorescence anisotropy indicated that the structural integrity of the constituent domains was maintained within the fusion protein. Furthermore, this engineered oligomerization function could be exploited to regulate protein–protein interactions in cellular contexts, shedding light on intricate signaling networks and cellular behavior. The engineered eCaM fusion protein provides a foundation for further research into creating intricate protein-based systems with Ex5 sequences at their N-termini, tailored for oligomerizing properties for diverse protein applications.

## Supporting Information Available

ASEC chromatogram profiles of CaM, Ex5, and a mixture of CaM and Ex5 and Ex5 and TRPM4 interaction analysis by FA (PDF)

## Abbreviations

AMBN: ameloblastin
ASEC: analytical size-exclusion chromatography
CaM: calmodulin
eCaM: engineered CaM protein composed of Ex5 peptide and CaM
Ex5: exon 5 peptide sequence derived from AMBN
IDP: intrinsically disordered protein
CBPs: calcium-binding proteins
TRP: transient receptor potential (channel)
TRPM4: TRP melastatin 4
DLS: dynamic light scattering
AUC: analytical ultracentrifugation
CD: circular dichroism
MDs: molecular dynamics simulations

## References

[ref1] Lutz S.; Iamurri S. M. Protein engineering: past, present, and future. In Protein Engineering: Methods and Protocols, 2018; pp 1–12. 10.1007/978-1-4939-7366-8_1 29086300

[ref2] Sinha R.; Shukla P. Current trends in protein engineering: updates and progress. Curr. Protein Pept. Sci. 2019, 20, 398–407. 10.2174/1389203720666181119120120. 30451109

[ref3] Ali M. H.; Imperiali B. Protein oligomerization: how and why. Bioorg. Med. Chem. 2005, 13, 5013–5020. 10.1016/j.bmc.2005.05.037. 15993087

[ref4] Kumari N.; Yadav S. Modulation of protein oligomerization: an overview. Prog. Biophys. Mol. Biol. 2019, 149, 99–113. 10.1016/j.pbiomolbio.2019.03.003. 30872157

[ref5] Gwyther R. E.; Jones D. D.; Worthy H. L. Better together: building protein oligomers naturally and by design. Biochem. Soc. Trans. 2019, 47, 1773–1780. 10.1042/BST20190283. 31803901 PMC6925524

[ref6] Gabizon R.; Friedler A. Allosteric modulation of protein oligomerization: an emerging approach to drug design. Front. Chem. 2014, 2, 9 10.3389/fchem.2014.00009. 24790978 PMC3982530

[ref7] Goodsell D. S.; Olson A. J. Structural symmetry and protein function. Annu. Rev. Biophys. Biomol. Struct. 2000, 29, 105–153. 10.1146/annurev.biophys.29.1.105. 10940245

[ref8] Levy E. D.; Teichmann S. A. Structural, evolutionary, and assembly principles of protein oligomerization. Prog. Mol. Biol. Transl. Sci. 2013, 117, 25–51. 10.1016/B978-0-12-386931-9.00002-7. 23663964

[ref9] Oohora K.; Hayashi T. Hemoprotein-based supramolecular assembling systems. Curr. Opin. Chem. Biol. 2014, 19, 154–161. 10.1016/j.cbpa.2014.02.014. 24658057

[ref10] Kobayashi N.; Arai R. Design and construction of self-assembling supramolecular protein complexes using artificial and fusion proteins as nanoscale building blocks. Curr. Opin. Biotechnol. 2017, 46, 57–65. 10.1016/j.copbio.2017.01.001. 28160725

[ref11] Ljubetič A.; Gradišar H.; Jerala R. Advances in design of protein folds and assemblies. Curr. Opin. Chem. Biol. 2017, 40, 65–71. 10.1016/j.cbpa.2017.06.020. 28709120

[ref12] Zakeri B.; Fierer J. O.; Celik E.; Chittock E. C.; Schwarz-Linek U.; Moy V. T.; Howarth M. Peptide tag forming a rapid covalent bond to a protein, through engineering a bacterial adhesin. Proc. Natl. Acad. Sci. U.S.A. 2012, 109, E690–E697. 10.1073/pnas.1115485109. 22366317 PMC3311370

[ref13] Brodin J. D.; Ambroggio X.; Tang C.; Parent K. N.; Baker T. S.; Tezcan F. A. Metal-directed, chemically tunable assembly of one-, two-and three-dimensional crystalline protein arrays. Nat. Chem. 2012, 4, 375–382. 10.1038/nchem.1290. 22522257 PMC3335442

[ref14] Jones D. D.; Barker P. D. Controlling Self-Assembly by Linking Protein Folding, DNA Binding, and the Redox Chemistry of Heme. Angew. Chem., Int. Ed. 2005, 44, 6337–6341. 10.1002/anie.200463035. 16163771

[ref15] Leibly D. J.; Arbing M. A.; Pashkov I.; DeVore N.; Waldo G. S.; Terwilliger T. C.; Yeates T. O. A suite of engineered GFP molecules for oligomeric scaffolding. Structure 2015, 23, 1754–1768. 10.1016/j.str.2015.07.008. 26278175 PMC4568552

[ref16] Fallas J. A.; Ueda G.; Sheffler W.; Nguyen V.; McNamara D. E.; Sankaran B.; Pereira J. H.; Parmeggiani F.; Brunette T.; Cascio D.; et al. Computational design of self-assembling cyclic protein homo-oligomers. Nat. Chem. 2017, 9, 353–360. 10.1038/nchem.2673. 28338692 PMC5367466

[ref17] Chevalier A.; Silva D.-A.; Rocklin G. J.; Hicks D. R.; Vergara R.; Murapa P.; Bernard S. M.; Zhang L.; Lam K.-H.; Yao G.; et al. Massively parallel de novo protein design for targeted therapeutics. Nature 2017, 550, 74–79. 10.1038/nature23912. 28953867 PMC5802399

[ref18] Gonen S.; DiMaio F.; Gonen T.; Baker D. Design of ordered two-dimensional arrays mediated by noncovalent protein-protein interfaces. Science 2015, 348, 1365–1368. 10.1126/science.aaa9897. 26089516

[ref19] Woolfson D. N. Coiled-coil design: updated and upgraded. Subcell Biochem. 2017, 82, 35–61. 10.1007/978-3-319-49674-0_2. 28101858

[ref20] Blondel A.; Bedouelle H. Engineering the quaternary structure of an exported protein with a leucine zipper. Protein Eng., Des. Sel. 1991, 4, 457–461. 10.1093/protein/4.4.457. 1881871

[ref21] Deisseroth K.; Heist E. K.; Tsien R. W. Translocation of calmodulin to the nucleus supports CREB phosphorylation in hippocampal neurons. Nature 1998, 392, 198–202. 10.1038/32448. 9515967

[ref22] Saimi Y.; Kung C. Calmodulin as an ion channel subunit. Annu. Rev. Physiol. 2002, 64, 289–311. 10.1146/annurev.physiol.64.100301.111649. 11826271

[ref23] Neil S. M.; Lakey T.; Tomlinson S. Calmodulin regulation of adenylate cyclase activity. Cell Calcium 1985, 6, 213–226. 10.1016/0143-4160(85)90007-7. 3893727

[ref24] Swulius M. T.; Waxham M. N. Ca(2+)/calmodulin-dependent protein kinases. Cell. Mol. Life Sci. 2008, 65, 2637–2657. 10.1007/s00018-008-8086-2. 18463790 PMC3617042

[ref25] Strynadka N. C.; James M. N. Crystal structures of the helix-loop-helix calcium-binding proteins. Annu. Rev. Biochem. 1989, 58, 951–998. 10.1146/annurev.bi.58.070189.004511. 2673026

[ref26] Kretsinger R. H.; Nockolds C. E. Carp muscle calcium-binding protein. II. Structure determination and general description. J. Biol. Chem. 1973, 248, 3313–3326. 10.1016/S0021-9258(19)44043-X. 4700463

[ref27] Babu Y. S.; Sack J. S.; Greenhough T. J.; Bugg C. E.; Means A. R.; Cook W. J. Three-dimensional structure of calmodulin. Nature 1985, 315, 37–40. 10.1038/315037a0. 3990807

[ref28] Babu Y. S.; Bugg C. E.; Cook W. J. Structure of calmodulin refined at 2.2 A resolution. J. Mol. Biol. 1988, 204, 191–204. 10.1016/0022-2836(88)90608-0. 3145979

[ref29] Barbato G.; Ikura M.; Kay L. E.; Pastor R. W.; Bax A. Backbone dynamics of calmodulin studied by 15N relaxation using inverse detected two-dimensional NMR spectroscopy: the central helix is flexible. Biochemistry 1992, 31, 5269–5278. 10.1021/bi00138a005. 1606151

[ref30] Lau S. Y.; Procko E.; Gaudet R. Distinct properties of Ca2+-calmodulin binding to N- and C-terminal regulatory regions of the TRPV1 channel. J. Gen. Physiol. 2012, 140, 541–555. 10.1085/jgp.201210810. 23109716 PMC3483115

[ref31] Ataman Z. A.; Gakhar L.; Sorensen B. R.; Hell J. W.; Shea M. A. The NMDA receptor NR1 C1 region bound to calmodulin: structural insights into functional differences between homologous domains. Structure 2007, 15, 1603–1617. 10.1016/j.str.2007.10.012. 18073110 PMC2246159

[ref32] Johnson C. N.; Potet F.; Thompson M. K.; Kroncke B. M.; Glazer A. M.; Voehler M. W.; Knollmann B. C.; George A. L.; Chazin W. J. A Mechanism of Calmodulin Modulation of the Human Cardiac Sodium Channel. Structure 2018, 26, 683–694.e3. 10.1016/j.str.2018.03.005. 29606593 PMC5932218

[ref33] Liu Y.; Zheng X.; Mueller G. A.; Sobhany M.; DeRose E. F.; Zhang Y.; London R. E.; Birnbaumer L. Crystal structure of calmodulin binding domain of orai1 in complex with Ca2+ calmodulin displays a unique binding mode. J. Biol. Chem. 2012, 287, 43030–43041. 10.1074/jbc.M112.380964. 23109337 PMC3522297

[ref34] Zhang M.; Abrams C.; Wang L. P.; Gizzi A.; He L. P.; Lin R. H.; Chen Y.; Loll P. J.; Pascal J. M.; Zhang J. F. Structural Basis for Calmodulin as a Dynamic Calcium Sensor. Structure 2012, 20, 911–923. 10.1016/j.str.2012.03.019. 22579256 PMC3372094

[ref35] Bahler M.; Rhoads A. Calmodulin signaling via the IQ motif. FEBS Lett. 2002, 513, 107–113. 10.1016/S0014-5793(01)03239-2. 11911888

[ref36] Vetyskova V.; Zouharova M.; Bednarova L.; Vaněk O.; Sázelová P.; Kašička V.; Vymetal J.; Srp J.; Rumlová M.; Charnavets T.; et al. Characterization of AMBN I and II isoforms and study of their Ca2+-binding properties. Int. J. Mol. Sci. 2020, 21, 9293 10.3390/ijms21239293. 33291486 PMC7730623

[ref37] Vetyskova V.; Hubalek M.; Sulc J.; Prochazka J.; Vondrasek J.; Vydra Bousova K. Proteolytic profiles of two isoforms of human AMBN expressed in E. coli by MMP-20 and KLK-4 proteases. Heliyon 2024, 10, e24564 10.1016/j.heliyon.2024.e24564. 38298721 PMC10828707

[ref38] Paine M. L.; Luo W.; Zhu D. H.; Bringas P.; Snead M. L. Functional domains for amelogenin revealed by compound genetic defects. J. Bone Miner. Res. 2003, 18, 466–472. 10.1359/jbmr.2003.18.3.466. 12619931 PMC12931981

[ref39] Lu X.; Li W.; Fukumoto S.; Yamada Y.; Evans C. A.; Diekwisch T.; Luan X. The ameloblastin extracellular matrix molecule enhances bone fracture resistance and promotes rapid bone fracture healing. Matrix Biol. 2016, 52–54, 113–126. 10.1016/j.matbio.2016.02.007. PMC5651174 26899203

[ref40] Su J.; Chandrababu K. B.; Moradian-Oldak J. Ameloblastin peptide encoded by exon 5 interacts with amelogenin N-terminus. Biochem. Biophys. Rep. 2016, 7, 26–32. 10.1016/j.bbrep.2016.05.007. 27725968 PMC5055063

[ref41] Wald T.; Osickova A.; Sulc M.; Benada O.; Semeradtova A.; Rezabkova L.; Veverka V.; Bednarova L.; Maly J.; Macek P.; Sebo P.; Slaby I.; Vondrasek J.; Osicka R. Intrinsically disordered enamel matrix protein ameloblastin forms ribbon-like supramolecular structures via an N-terminal segment encoded by exon 5. J. Biol. Chem. 2013, 288, 22333–22345. 10.1074/jbc.M113.456012. 23782691 PMC3829324

[ref42] Wald T.; Spoutil F.; Osickova A.; Prochazkova M.; Benada O.; Kasparek P.; Bumba L.; Klein O. D.; Sedlacek R.; Sebo P.; et al. Intrinsically disordered proteins drive enamel formation via an evolutionarily conserved self-assembly motif. Proc. Natl. Acad. Sci. U.S.A. 2017, 114, E1641–E1650. 10.1073/pnas.1615334114. 28196895 PMC5338493

[ref43] Su J.; Kegulian N. C.; Bapat R. A.; Moradian-Oldak J. Ameloblastin binds to phospholipid bilayers via a helix-forming motif within the sequence encoded by exon 5. ACS Omega 2019, 4, 4405–4416. 10.1021/acsomega.8b03582. 30873509 PMC6410667

[ref44] Lu X.; Ito Y.; Kulkarni A.; Gibson C.; Luan X.; Diekwisch T. G. Ameloblastin-rich enamel matrix favors short and randomly oriented apatite crystals. Eur. J. Oral Sci. 2011, 119, 254–260. 10.1111/j.1600-0722.2011.00905.x. 22243254 PMC3402546

[ref45] Ravindranath H. H.; Chen L.-S.; Zeichner-David M.; Ishima R.; Ravindranath R. M. Interaction between the enamel matrix proteins amelogenin and ameloblastin. Biochem. Biophys. Res. Commun. 2004, 323, 1075–1083. 10.1016/j.bbrc.2004.08.207. 15381109

[ref46] Pandya M.; Diekwisch T. G. Amelogenesis: Transformation of a protein-mineral matrix into tooth enamel. J. Struct. Biol. 2021, 213, 107809 10.1016/j.jsb.2021.107809. 34748943 PMC8665087

[ref47] Kegulian N. C.; Visakan G.; Bapat R. A.; Moradian-Oldak J. Ameloblastin and its multifunctionality in amelogenesis: a review. Matrix Biol. 2024, 131, 62 10.1016/j.matbio.2024.05.007. 38815936 PMC11218920

[ref48] Bousova K.; Herman P.; Vecer J.; Bednarova L.; Monincova L.; Majer P.; Vyklicky L.; Vondrasek J.; Teisinger J. Shared CaM- and S100A1-binding epitopes in the distal TRPM4 N terminus. FEBS J. 2018, 285, 599–613. 10.1111/febs.14362. 29240297

[ref49] Hayes D.; Laue T.; Philo J. Program Sednterp: Sedimentation Interpretation Pprogram; Alliance Protein Laboratories: Thousand Oaks, CA, 1995.

[ref50] Schuck P. Size-distribution analysis of macromolecules by sedimentation velocity ultracentrifugation and lamm equation modeling. Biophys. J. 2000, 78, 1606–1619. 10.1016/S0006-3495(00)76713-0. 10692345 PMC1300758

[ref51] Brautigam C. A. Calculations and Publication-Quality Illustrations for Analytical Ultracentrifugation Data. Methods Enzymol. 2015, 562, 109–133. 10.1016/bs.mie.2015.05.001. 26412649

[ref52] Sreerama N.; Woody R. W. Estimation of protein secondary structure from circular dichroism spectra: comparison of CONTIN, SELCON, and CDSSTR methods with an expanded reference set. Anal. Biochem. 2000, 287, 252–260. 10.1006/abio.2000.4880. 11112271

[ref53] Bousova K.; Barvik I.; Herman P.; Hofbauerová K.; Monincova L.; Majer P.; Zouharova M.; Vetyskova V.; Postulkova K.; Vondrasek J. Mapping of CaM, S100A1 and PIP2-Binding Epitopes in the Intracellular N-and C-Termini of TRPM4. Int. J. Mol. Sci. 2020, 21, 4323 10.3390/ijms21124323. 32560560 PMC7352223

[ref54] Wald T.; Bednarova L.; Osicka R.; Pachl P.; Sulc M.; Lyngstadaas S. P.; Slaby I.; Vondrasek J. Biophysical characterization of recombinant human ameloblastin. Eur. J. Oral Sci. 2011, 119 (s1), 261–269. 10.1111/j.1600-0722.2011.00913.x. 22243255

[ref55] Martin S. R.; Bayley P. M. The effects of Ca2+ and Cd2+ on the secondary and tertiary structure of bovine testis calmodulin. A circular-dichroism study. Biochem. J. 1986, 238, 485–490. 10.1042/bj2380485. 3800949 PMC1147160

[ref56] Hashimoto K.; Panchenko A. R. Mechanisms of protein oligomerization, the critical role of insertions and deletions in maintaining different oligomeric states. Proc. Natl. Acad. Sci. U.S.A. 2010, 107, 20352–20357. 10.1073/pnas.1012999107. 21048085 PMC2996646

[ref57] Frieden C. Protein oligomerization as a metabolic control mechanism: Application to apoE. Protein Sci. 2019, 28, 837–842. 10.1002/pro.3583. 30701627 PMC6423707

[ref58] Strotmann R.; Schultz G.; Plant T. D. Ca2+-dependent potentiation of the nonselective cation channel TRPV4 is mediated by a C-terminal calmodulin binding site. J. Biol. Chem. 2003, 278, 26541–26549. 10.1074/jbc.M302590200. 12724311

[ref59] Tong Q.; Zhang W.; Conrad K.; Mostoller K.; Cheung J. Y.; Peterson B. Z.; Miller B. A. Regulation of the transient receptor potential channel TRPM2 by the Ca2+ sensor calmodulin. J. Biol. Chem. 2006, 281, 9076–9085. 10.1074/jbc.M510422200. 16461353

[ref60] Numazaki M.; Tominaga T.; Takeuchi K.; Murayama N.; Toyooka H.; Tominaga M. Structural determinant of TRPV1 desensitization interacts with calmodulin. Proc. Natl. Acad. Sci. U.S.A. 2003, 100, 8002–8006. 10.1073/pnas.1337252100. 12808128 PMC164702

[ref61] Rosenbaum T.; Gordon-Shaag A.; Munari M.; Gordon S. E. Ca2+/calmodulin modulates TRPV1 activation by capsaicin. J. Gen. Physiol. 2004, 123, 53–62. 10.1085/jgp.200308906. 14699077 PMC2217413

[ref62] Xiao R.; Tang J.; Wang C.; Colton C. K.; Tian J.; Zhu M. X. Calcium plays a central role in the sensitization of TRPV3 channel to repetitive stimulations. J. Biol. Chem. 2008, 283, 6162–6174. 10.1074/jbc.M706535200. 18178557 PMC2287377

[ref63] de Groot T.; Kovalevskaya N. V.; Verkaart S.; Schilderink N.; Felici M.; van der Hagen E. A.; Bindels R. J.; Vuister G. W.; Hoenderop J. G. Molecular mechanisms of calmodulin action on TRPV5 and modulation by parathyroid hormone. Mol. Cell. Biol. 2011, 31, 2845–2853. 10.1128/MCB.01319-10. 21576356 PMC3133394

[ref64] Derler I.; Hofbauer M.; Kahr H.; Fritsch R.; Muik M.; Kepplinger K.; Hack M. E.; Moritz S.; Schindl R.; Groschner K.; Romanin C. Dynamic but not constitutive association of calmodulin with rat TRPV6 channels enables fine tuning of Ca2+-dependent inactivation. J. Physiol. 2006, 577, 31–44. 10.1113/jphysiol.2006.118661. 16959851 PMC2000671

[ref65] Holakovska B.; Grycova L.; Bily J.; Teisinger J. Characterization of calmodulin binding domains in TRPV2 and TRPV5 C-tails. Amino Acids 2011, 40, 741–748. 10.1007/s00726-010-0712-2. 20686800

[ref66] Zouharova M.; Herman P.; Hofbauerova K.; Vondrasek J.; Bousova K. TRPM6 N-Terminal CaM- and S100A1-Binding Domains. Int. J. Mol. Sci. 2019, 20, 4430 10.3390/ijms20184430. 31505788 PMC6770577

[ref67] Holakovska B.; Grycova L.; Jirku M.; Sulc M.; Bumba L.; Teisinger J. Calmodulin and S100A1 protein interact with N terminus of TRPM3 channel. J. Biol. Chem. 2012, 287, 16645–16655. 10.1074/jbc.M112.350686. 22451665 PMC3351314

[ref68] Gloss L. M. Equilibrium and kinetic approaches for studying oligomeric protein folding. Methods Enzymol. 2009, 325–357. 10.1016/s0076-6879(09)66014-6. 21609867

[ref69] Paul S. S.; Lyons A.; Kirchner R.; Woodside M. T. Quantifying oligomer populations in real time during protein aggregation using single-molecule mass photometry. ACS Nano 2022, 16, 16462–16470. 10.1021/acsnano.2c05739. 36126253 PMC9620981

