# Supplementary material for: Oligomerization Function of the Native Exon 5 Sequence of Ameloblastin Fused with Calmodulin
Source: ACS Omega. 2025 Feb 20;10(8):7741–51. doi: 10.1021/acsomega.4c07953 (PMC11886713; doi:10.1021/acsomega.4c07953)
Supplement: Supplementary file 1 — ao4c07953_si_001.pdf [file ao4c07953_si_001.pdf]

Oligomerization function of native exon 5 sequence of ameloblastin fused with calmodulin

Monika Zouharova [a, b], Petr Herman [c], Lucie Bednarova [a], Veronika Vetyskova [a], Romana Hadravova [a], Klara Postulkova [a], Lucie Zemanova [d], Jiri Vondrasek [a, *] and Kristyna Vydra Bousova [a, *]

[a] Institute of Organic Chemistry and Biochemistry of the Czech Academy of Sciences, Flemingovo namesti 5/542, 16000, Prague, Czech Republic

[b] Charles University, Second Faculty of Medicine, V Úvalu 84, 15006, Prague, Czech Republic

[c] Charles University, Faculty of Mathematics and Physics, Ke Kralovu 5, 12116, Prague, Czech Republic

[d] University of Hradec Kralove, Faculty of Science, Rokitanskeho 62, 500 03, Hradec Kralove, Czech Republic

* Corresponding author:
Kristyna Vydra Bousova, Jiri Vondrasek
E-mail: kristyna.bousova@uochb.cas.cz, jiri.vondrasek@uochb.cas.cz
Telephone: 00420220183131
Address: Institute of Organic Chemistry and Biochemistry, CAS
Flemingovo namesti 2
Prague 16000, Czech Republic

Supplementary material

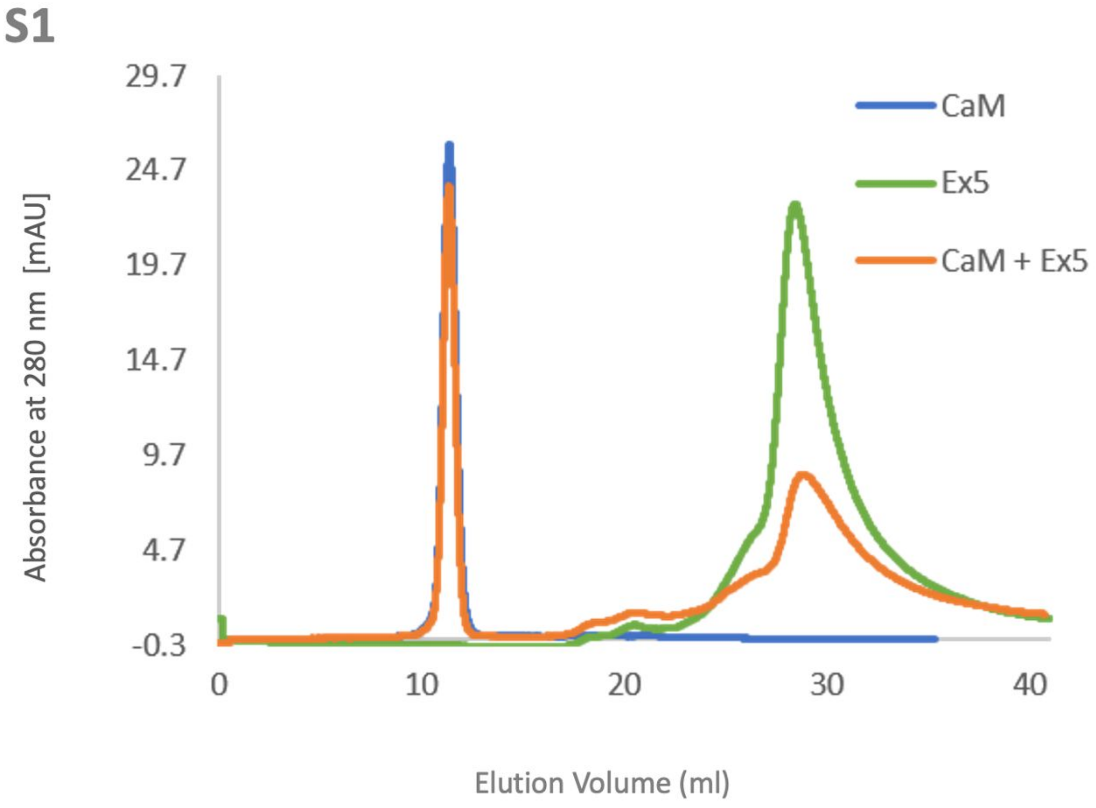

S1 ASEC chromatogram profiles of CaM (blue), Ex5 (green) and mixture of CaM and Ex5 (orange). Proteins were analysed at 2.56 mg/ml concentrations in Day 1 indicating no interaction of separated CaM and Ex5 in the mixture. All protein batches were eluted on a Superdex 200 Increase 10/300 GL column on Acta Pure facility.

S2

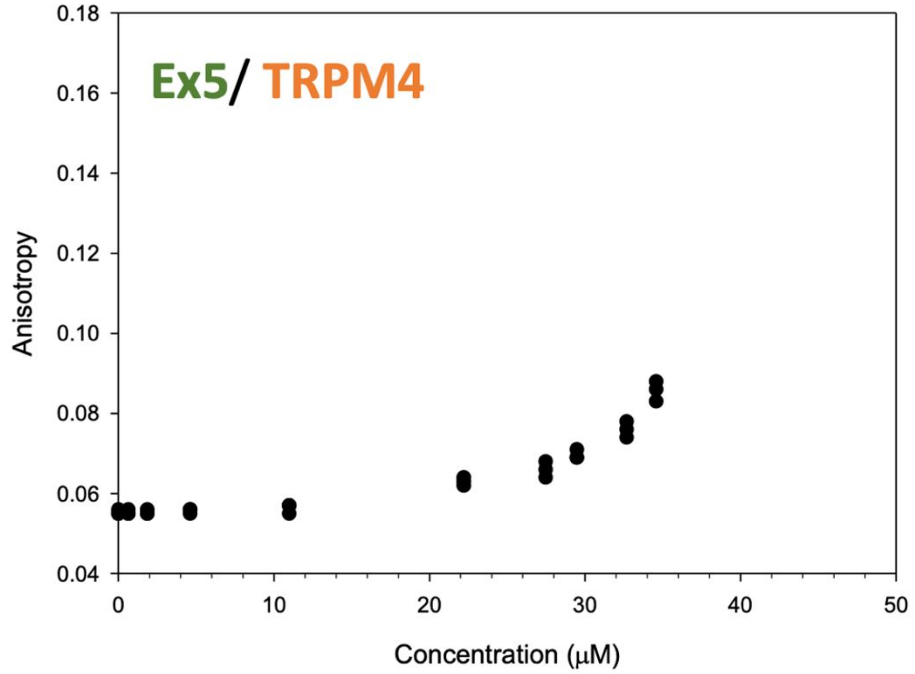

S2 Ex5 and TRPM4 interaction analysis by FA. (A) The anisotropy of the TRPM4np as a function of Ex5 peptide concentration obtained from steady-state fluorescence anisotropy titrations indicated no interaction.
